# Manual of Operative Surgery

**Published:** 1887-03

**Authors:** 


**Manual of Operative Surgery.** By JOSEPH D. BRYANT, M. D., Professor of Anatomy and Clinical Surgery and Associate Professor of Orthopedic Surgery, Bellevue Hospital Medical College; Visiting Surgeon to Bellevue Hospital; Consulting Surgeon to the Bureau of Medical and Surgical Relief of Bellevue Hospital; Consulting Surgeon to the New York Lunatic Asylum and to the Northwestern Dispensary. With about eight hundred illustrations. New York: D. Appleton & Co. 1887.

This volume is very properly dedicated to Dr. Stephen Smith, of New York, who was the author of the first hand-book of operative surgery. The two books are quite similar in scope. This is somewhat larger and on certain subjects more exhaustive. It might be safe to say that a physician would find both of great usefulness to him. The cuts are most excellent, and the general style of the book neat. In the first chapter, the author makes some most excellent suggestions as to facts to be ascertained before operation, the preparation of the patient, diet and essential requirements. The second chapter is on the controlling of hemorrhage, and here, too, are introduced many novel and useful hints. The third and fourth are on "treatment of operated wounds" and "ligature of arteries." Through all the eighteen chapters of the book many new and valuable suggestions are made. As we all know Dr. Bryant is eminently a successful teacher, we should expect such a book from him useful to students and physicians.

